# Diagnostic assessment of artificial intelligence reconstruction on accelerated prostate MRI: a retrospective, paired, multi-reader multi-case study

**DOI:** 10.1007/s00330-026-12479-7

**Published:** 2026-04-02

**Authors:** Quintin van Lohuizen, Stefan Johannes Fransen, George Yiasemis, Jasper Jonathan Twilt, Christian Roest, Yuki Arita, Jacob Borstlap, Jurgen J. Fütterer, Maarten de Rooij, Dennis B. Rouw, Ivo G. Schoots, Baris Turkbey, Samuel Joseph Withey, Frank F. J. Simonis, Henkjan Huisman, Thomas Christian Kwee, Jonas Teuwen, Derya Yakar

**Affiliations:** 1 Department of Radiology, University Medical Center Groningen, Groningen, The Netherlands; 2 Department of Radiation Oncology, The Netherlands Cancer Institute, Amsterdam, The Netherlands; 3 Department of Medical Imaging, Radboud University Medical Center, Nijmegen, The Netherlands; 4 Memorial Sloan Kettering Cancer Center, New York, NY USA; 5 Department of Radiology, Treant Hospital, Emmen, The Netherlands; 6 Martini Ziekenhuis, Groningen, The Netherlands; 7 Department of Radiology & Nuclear Medicine, Erasmus University Medical Center, Rotterdam, The Netherlands; 8 Molecular Imaging Branch, National Cancer Institute, National Institutes of Health, Bethesda, MD USA; 9 Department of Radiology, The Royal Marsden NHS Foundation Trust, London, UK; 10 Magnetic Detection and Imaging, Technical Medical Centre, University of Twente, Enschede, The Netherlands; 11 Department of Radiology, The Netherlands Cancer Institute, Amsterdam, The Netherlands

**Keywords:** Magnetic resonance imaging, Image reconstruction, Deep learning, Prostatic neoplasms, Diagnostic imaging

## Abstract

**Objectives:**

To determine whether AI-reconstructed prostate MRI at reduced acquisition times maintains prostate cancer (PCa) detection performance comparable to conventional scans.

**Materials and methods:**

This multicenter, retrospective, consecutive-cohort study included 120 multi-coil T2-weighted prostate MRI scans from the University Medical Center Groningen (UMCG) and 312 publicly available scans from New York University (NYU). An AI model trained on the NYU data was tested on retrospectively undersampled UMCG scans at acceleration factors R = 3 and R = 6 (i.e., data reduction in k-space). Eight experienced radiologists participated in a multi-reader multi-case PCa detection study. Diagnostic performance was assessed using the area under the receiver operating characteristic curve (AUROC). Histopathology and PI-RADS ≤ 2 findings served as reference standards. Multiple image quality metrics were subjectively evaluated using a 4-point Likert scale.

**Results:**

No statistically significant reduction in PCa detection was observed at an MRI acceleration up to R = 6 (*p* = 0.08). AUROC values were 0.86 (95% CI: 0.74–0.90) for R = 1, 0.82 (0.72–0.88) for R = 3, and 0.80 (0.70–0.86) for R = 6. Compared to R = 1, R = 3 scans were rated by radiologists to have significantly improved sharpness (+0.2, *p* < 0.05) and lower noise (+0.1, *p* < 0.05). Overall visual quality at R = 6 remained comparable to R = 1 (2.81 at R6 vs. 2.74 at R1).

**Conclusion:**

AI-driven reconstruction enabled a sixfold acceleration of T2-weighted prostate MRI (0:33–1:27 min) without a statistically significant reduction in PCa detection, while preserving perceived image quality. However, the decreasing diagnostic performance at higher accelerations warrants further prospective evaluation.

**Key Points:**

***Question*** *This study investigated whether deep learning reconstruction enables three- to sixfold acceleration without reducing radiologists’ detection of clinically significant prostate cancer*.

***Findings*** *In a multi-reader multi-case study with eight radiologists, three- and sixfold acceleration showed no significant change in area under the receiver operating characteristic curve*.

***Clinical relevance*** *Deep learning reconstruction shortened T2-weighted acquisition times at sixfold acceleration while preserving perceived image quality and diagnostic performance across acceleration factors*.

**Graphical Abstract:**

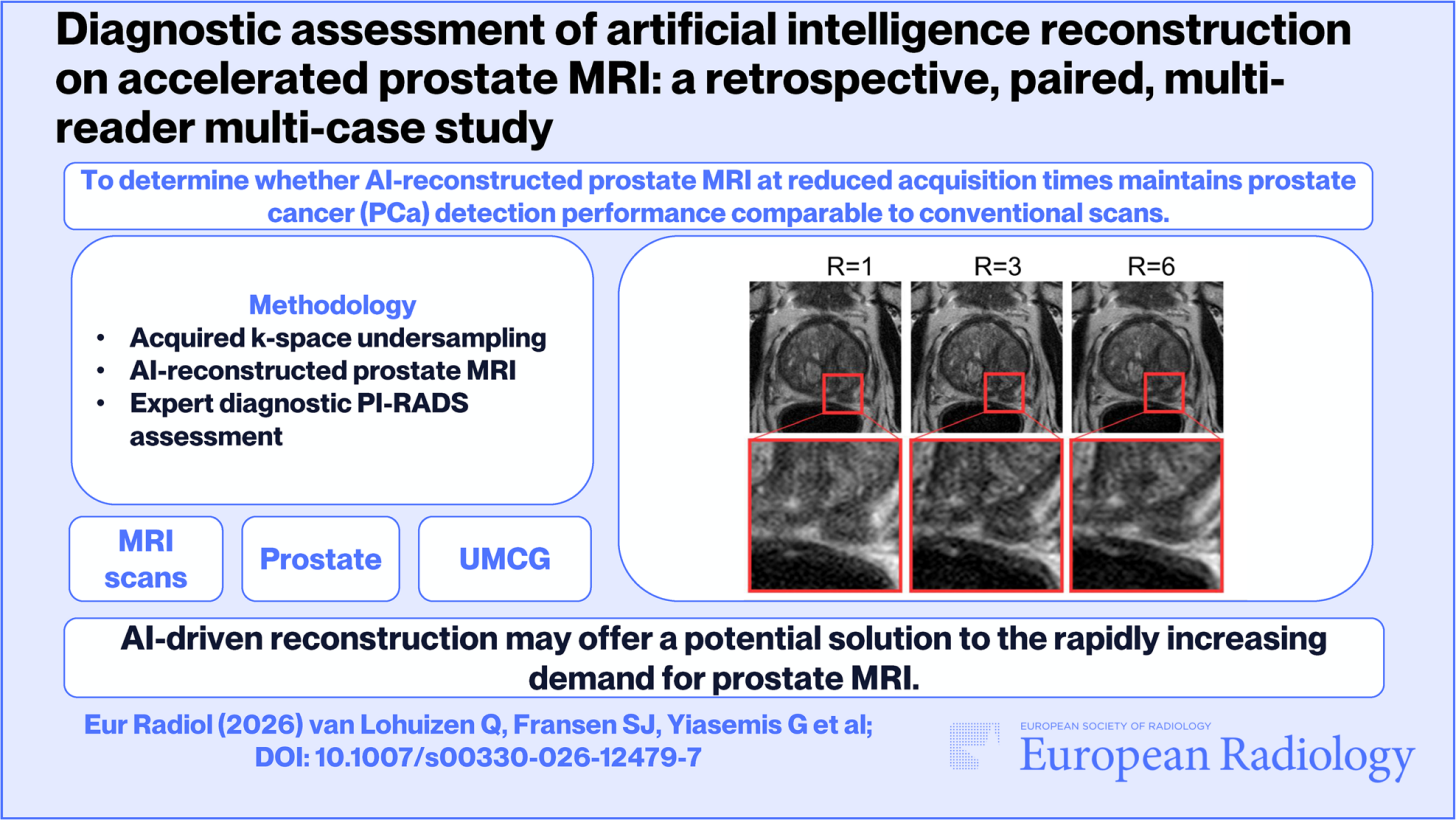

## Introduction

MRI is essential for diagnosing and staging various cancers, including clinically significant prostate cancer (csPCa), and T2-weighted imaging is central for transition-zone lesion assessment and local staging. However, conventional MRI protocols have long acquisition times, making it challenging to meet the rapidly increasing demand [1]. Although conventional acceleration techniques like parallel imaging and compressed sensing can reduce scan times, they often compromise image quality at high acceleration factors and are fundamentally limited by physical constraints. Recent advancements in MRI acceleration, such as data undersampling combined with artificial intelligence (AI) reconstruction, have substantially reduced scan times and improved image quality at higher acceleration factors compared to standard imaging [2–5].

While AI-based reconstruction methods have achieved notable improvements in computed visual quality metrics compared with conventional techniques [2–5], their impact on clinical diagnostic performance remains insufficiently explored. Moreover, most evaluations to date have relied on simulated or single-coil data, which are highly valuable for algorithm development but may not fully reflect the complexity of real-world, multi-coil imaging [5]. Validation under clinical conditions with expert radiologists and biopsy reference standards remains essential to establish diagnostic reliability [4, 6].

This study aimed to assess whether AI-reconstructed T2-weighted (T2w) MRI scans at three- and six-times acceleration could achieve diagnostic performance comparable to conventional non-accelerated imaging. A multi-reader, multi-case evaluation was conducted using clinically acquired, multi-coil T2w prostate MRI interpreted by eight experienced prostate radiologists.

## Materials and methods

### Training data

The publicly available fastMRI prostate dataset from NYU (New York University, training dataset) [7] was used for model development and training under an institutional data-sharing agreement. The NYU dataset was selected for its multi-coil data acquisitions, large sample size, and public accessibility. It included 312 scans with T2w axial images and diffusion-weighted imaging (DWI). Detailed acquisition parameters for the NYU dataset are provided in Table 1.

**Table 1:** T2-weighted axial acquisition parameters

T2w parameters	R = 1	R = 1	R = 3	R = 6
Institute	NYU	UMCG		
Siemens MR type	Vida	Skyra (64%)^a^, Prisma (36%)^a^		
Field strength	3 T	3 T		
Parallel imaging factor	2	2	2	4
Averages, *n*	3	3	1	1
Scan time (min:s)	1:35–3:14^b^	3:18–8:40 IQR: 31 s	1:06–2:53^b^	0:33–1:27^b^
Phase encoding steps	451	Skyra: 551, Prisma: 651		
Voxel size (mm × mm)	0.56 × 0.56	Skyra: 0.56 × 0.56, Prisma: 0.47 × 0.47		
Matrix size	320 × 320	Skyra: 320 × 256, Prisma: 384 × 307		
Slice thickness (mm)	3	3		
FOV (mm × mm)	180 × 180	180 × 180		
TR (msec)	3500–7200	3640–13,700		
TE (msec)	100	103–118		
Coils, *n*	10–30	10–30		
Slices, *n*	30–36	26–38		

Acquisition parameters of T2w axial training and testing data are shown. An acceleration of R = 3 was achieved by using one average, while R = 6 also used a single average with a doubled parallel imaging factor. Estimated scan times are based on k-space acquisition lines and TR/TE values. Ranges are provided for parameters with patient-dependent variability. Intra-patient scan times were calculated with retrospective acquisition parameters based on the undersampling rate

*FOV* field of view, *TE* echo time, *TR* repetition time, *R-value* relative acceleration factor to reference standard

^a^ Skyra (64%) and Prisma (36%) proportions reflect scanner distribution

^b^ Intra-patient calculated scan time

### Testing data

Ethical approval for the testing dataset was obtained from the institutional review board of the University Medical Center Groningen (UMCG, testing dataset) (METc 2022/082), which waived the need for informed consent. The initial consecutive cohort included 157 multi-coil biparametric MRI scans acquired during routine clinical practice between May 2022 and January 2024. These scans were obtained from patients with clinical suspicion of csPCa. Each visit included T2w axial images, DWI with calculated b1400, and an apparent diffusion coefficient map (ADC). Exclusion criteria included prior prostate treatment (e.g., proctectomy and transurethral resection of the prostate), PI-RADS ≥ 3 without follow-up biopsy, follow-up examinations of the same patient, scans performed for nondiagnostic purposes, and missing T2w images. Only baseline scans were retained to ensure case independence and avoid repeated measures.

### Reference standard

The clinically acquired, non-accelerated T2w MRI served as the reference standard for both diagnostic and image quality assessments. All reference standard examinations were prospectively interpreted in routine clinical practice by at least one of six radiologists (5–10 years of experience) at UMCG, in accordance with PI-RADS guidelines [8]. Patients with PI-RADS ≥ 3 lesions underwent targeted biopsies; systematic biopsies were selectively performed. Our study defined csPCa positive cases as GGG = 2–5 (Gleason scores 7–10, intermediate to very high risk) and negative cases as PI-RADS ≤ 2 and/or biopsy-negative findings (e.g., GGG = 1 (Gleason score 6; low risk)). For the image-based reference standard, the clinically acquired multi-coil biparametric MRI was reconstructed using the conventional root-sum-of-squares method. The multi-coil T2w data were extracted directly from the clinical pipeline just before the vendor-specific reconstruction step, ensuring consistency with standard clinical practice. Following CLAIM guidelines, further methodological details are provided in Supplementary Material 1.4 [9].

### Data acquisition and undersampling

During routine clinical practice at the UMCG, we collected consecutive multi-coil biparametric MRI datasets, including raw k-space and DICOM images. These data are typically reconstructed with a baseline parallel imaging factor of two (see Table 1). Acceleration factor R denotes the degree of k-space undersampling relative to the fully sampled reference acquisition (R = 1). In this study, the reference standard (R = 1, 3:18–8:40 min) was retrospectively undersampled to simulate accelerated scans at R = 3 (1:06–2:53 min) and R = 6 (0:33–1:27 min): R = 3 was achieved by acquiring one of three signal averages, and R = 6 by combining a single average with a doubled parallel imaging factor (see Table 1) [5]. These acceleration factors were selected for their practical feasibility within standard scanner configurations. The DWI was not undersampled (Skyra (64%) 3:21–3:42 min, Prisma (36%) 2:33–2:58 min; Supplementary Materials, Table S2). This approach enables evaluation of clinically feasible scan accelerations using routine acquisition modifications, while assessing their impact on diagnostic image quality and prostate cancer detection.

### Model development and training protocols

For accelerated reconstruction, we used the vSHARP model, a state-of-the-art AI method designed to reconstruct high-quality images from undersampled k-space data [10]. Its effectiveness was demonstrated in the Cardiac MRI Reconstruction Challenge at MICCAI 2023, where it ranked first in inference speed and second in computed image quality [11, 12]. The model integrates deep learning priors with physics-based data consistency through an iterative optimization framework. For this study, vSHARP was retrained de novo on T2w prostate MRI data to enable domain-specific reconstruction. Further details on the data characteristics, model parameters, and performance metrics are provided in Supplementary Material 1.2.

### Reader study design

To evaluate the diagnostic performance of AI-based accelerated prostate MRI reconstructions across acceleration factors (R = 1, R = 3, R = 6), a reader study design was chosen that optimized the workload per reader and ensured sufficient statistical power. A partially paired, split-plot multi-reader multi-case (MRMC) [13, 14] study was conducted with 120 consecutive prostate MRIs from the testing dataset. The sample size selection was informed by a power analysis using the iMRMC tool [15]. The power analysis ensured sufficient statistical power (≥ 80%) to detect differences in diagnostic accuracy across readers and cases, accounting for observer variability, target accuracy levels, and expected performance differences (see Supplementary Material 1.1 for details).

To manage workload and ensure a balanced case distribution, the dataset and radiologists were divided into three groups, each containing 40 cases. Radiologists participated in three consecutive reading sessions, during which they evaluated a randomly stratified set of 40 scans per session, with acceleration conditions evenly distributed (R = 1, R = 3, R = 6). Sessions were spaced 4 weeks apart to avoid recall bias. Within each session, radiologists first assessed the T2w image for visual quality metrics and diagnostic metrics, including csPCa suspicion scores and PI-RADS assessments. They then immediately repeated the diagnostic assessment for the same case with the standard, non-accelerated DWI and ADC without the possibility of revisiting or altering their initial T2w assessments. Full protocol details and workflow are described in Supplementary Material 1.3. All readings were conducted on the online GrandChallenge platform.

### Statistical analysis

Statistical analysis compared diagnostic and image quality outcomes across reconstruction conditions (R = 1, R = 3, R = 6). The primary endpoint was diagnostic performance, assessed using reader-level area under the receiver operating characteristic curve (AUROC) derived from continuous csPCa suspicion scores and compared across conditions using an MRMC analysis. Secondary endpoints included perceived visual quality Likert ratings and objective image similarity metrics. Perceived visual quality ratings were analyzed using an ordinal mixed-effects model. A significance level of *p* < 0.05 was applied throughout.

### Diagnostic performance assessment

The primary endpoint was diagnostic performance across reconstruction conditions, evaluated using an MRMC study design. Eight radiologists independently rated patient-level suspicion of csPCa on a continuous scale (0–100). AUROC values were computed per reader and averaged using an area-preserving approach [16]. Confidence intervals were calculated using 10,000 bootstrap resamples of patients with replacement while keeping the participating readers fixed. These confidence intervals reflect case-level sampling uncertainty. Variance decomposition for MRMC analysis was performed using the MRMCAov R package [13, 14, 17]. Lesion-level sensitivity and specificity were assessed by classifying each reader-identified csPCa lesion (PI-RADS ≥ 3) as a ‘hit’ or ‘miss’ based on its location relative to biopsy-confirmed csPCa (Gleason grade group ≥ 2), allowing a 5 mm margin to accommodate spatial uncertainty [14, 18]. Inter-reader agreement within each reconstruction condition was quantified via intraclass correlation coefficients (ICC), using a one-way random-effects model for average measures. A significance threshold of *p* < 0.05 was applied. Additional details on power analysis, reader study protocol, and evaluation metrics are provided in Supplementary Material 1.1, 1.3, and 1.4, respectively.

### Visual performance assessment

The secondary endpoint evaluated image quality differences between AI-reconstructed and non-accelerated reference images using PI-QUALv2-derived [19] metrics. Radiologists rated artifacts, sharpness, noise, lesion conspicuity, and overall image quality on a four-point Likert scale, from nondiagnostic (1) to perfect diagnostic quality (4). Differences were assessed using an ordinal mixed-effects model [20] with acceleration condition and question as fixed effects and reader and patient as random effects. Objective image quality was evaluated using the structural similarity index measure, peak signal-to-noise ratio, root mean square error, and high-frequency error norm, quantifying perceptual similarity, overall noise, reconstruction error, and fine-detail preservation, respectively [21, 22]. Additional details on visual metrics and evaluation procedures are provided in Supplementary Material 1.4.4.

## Results

### Testing data

**Fig. 1:**
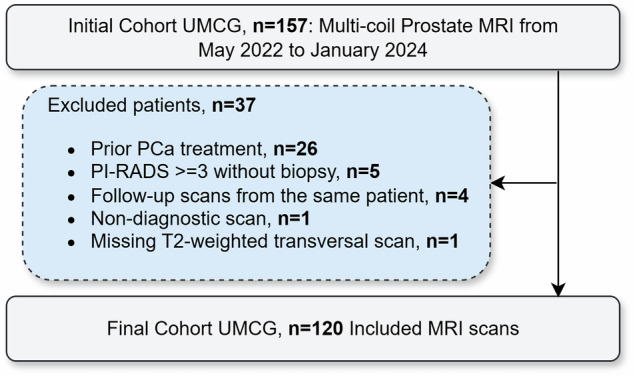
STARD diagram of the test dataset. The independent testing dataset consisted of 157 consecutive multi-coil prostate MRI scans acquired during routine clinical practice at UMCG between May 2022 and January 2024. These scans, intended for clinical use, were retrospectively analyzed for this study. MRI, magnetic resonance imaging; PCa, prostate cancer; PI-RADS, prostate imaging-reporting and data systems

**Fig. 2:**
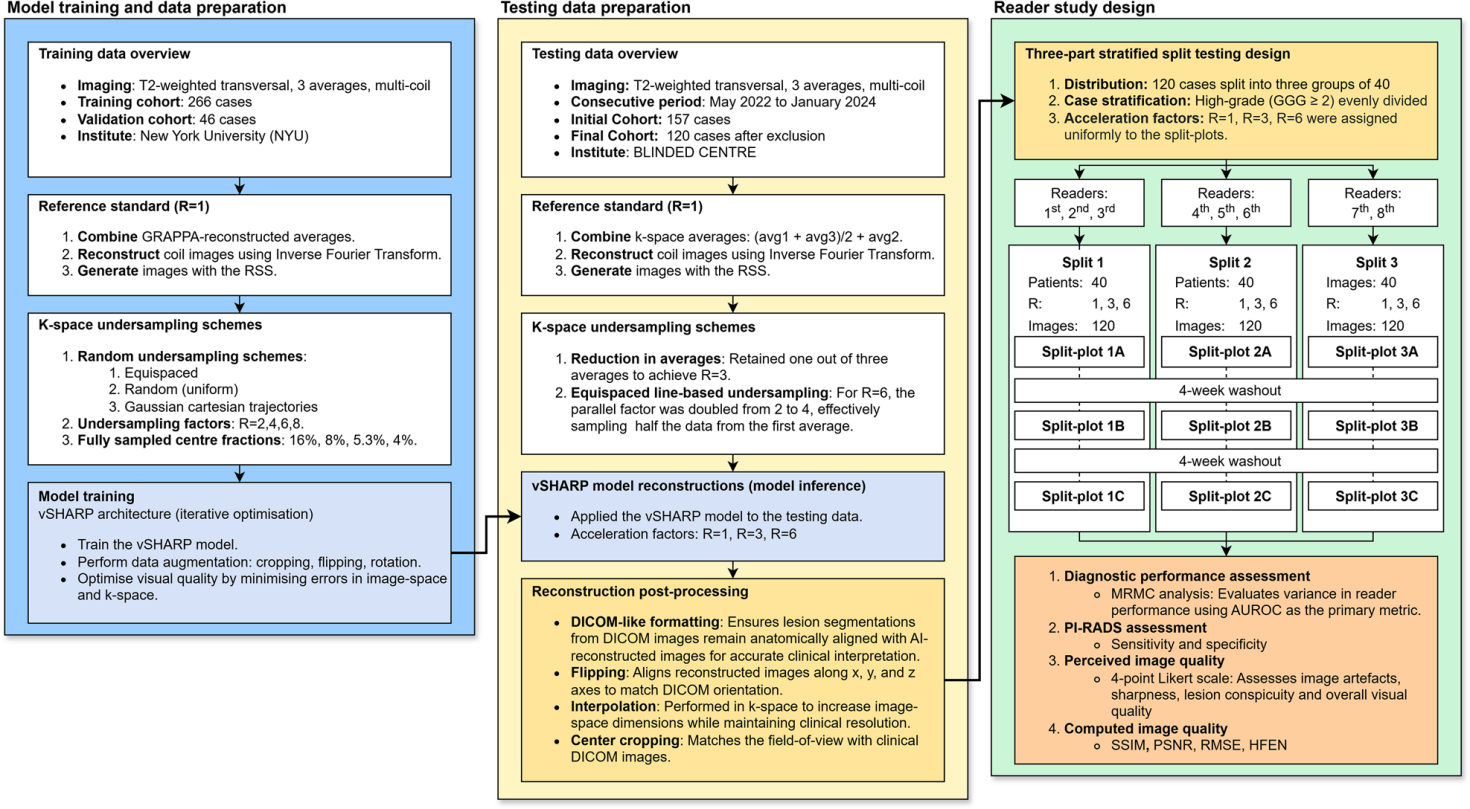
Methodological assessment framework. This framework outlines the methodology for training, validating, and testing a multi-coil prostate MRI reconstruction model. The left panel covers training data preparation, the center panel details testing and preprocessing steps, and the right panel presents diagnostic and visual quality evaluations. Acceleration factors (R = 1, R = 3, R = 6) were tested. DICOM, digital imaging and communications in medicine; T2w, T2-weighted imaging; GRAPPA, generalized autocalibrating partially parallel acquisitions; RSS, root sum of squares; R, undersampling factor; GGG, Gleason grade group; MRMC, multi-reader multi-case; PI-RADS, prostate imaging-reporting and data system; SSIM, structural similarity index measure; PSNR, peak signal-to-noise ratio; RMSE, root mean square error; HFEN, high-frequency error norm

Detailed patient inclusion is visualized in Fig. 1. A total of 120 patients were included, of which 49 patients had a prostate imaging reporting and data systems score (PI-RADS) ≥ 3, and 25 patients had a Gleason grade group (GGG) ≥ 2. Negative cases were primarily based on PI-RADS ≤ 2 (71 cases (75%)) and the remainder on histopathology (24 cases (25%)). The median follow-up duration between MRI scan and biopsy for all cases was 1.4 (IQR 0.7–1.9) months, for negative cases was 1.3 (0.6–2.0) months, and for positive cases was 1.4 (0.9–1.8) months. The patients had a mean age of 68.1 ± 8.0 years, prostate-specific antigen level of 10.0 ± 9.3 ng/mL, and prostate-specific antigen density level of 0.22 ± 0.20 ng/mL/cc (Table 2). Detailed data processing can be found in Fig. 2. T2w acquisition parameters are described in Table 1. An overview of DWI parameters and the size and prostate region of the lesions are described in the Supplementary Tables S2 and S3, respectively.

**Table 2:** Testing data patient demographics

Patients, *n*	120
Patients (PI-RADS ≥ 3), *n*	49
Patients (GGG ≥ 2), *n*	25
Age (years)*	68.1 ± 8.0
PSA (ng/mL)*	10.0 ± 9.3 (*n* = 111)
PSAD (ng/mL/cc)*	0.22 ± 0.20 (*n* = 108)

Here, we present the patient characteristics of the UMCG testing dataset

*GGG* Gleason grade group, *PI-RADS* prostate imaging-reporting and data systems, *PSA* prostate-specific antigen, *PSAD* prostate-specific antigen density

* Mean ± standard deviation

### Readers

Eight radiologists with a mean of 12.1 ± 4.3 years of prostate MRI experience participated. All had interpreted over 1000 clinical prostate MRI cases. Readers were based in the Netherlands (*n* = 5), the United States (*n* = 2), and the United Kingdom (*n* = 1). Further details are provided in Supplementary Material 2.1.

### Diagnostic performance assessment

#### MRMC detection analysis

**Fig. 3:**
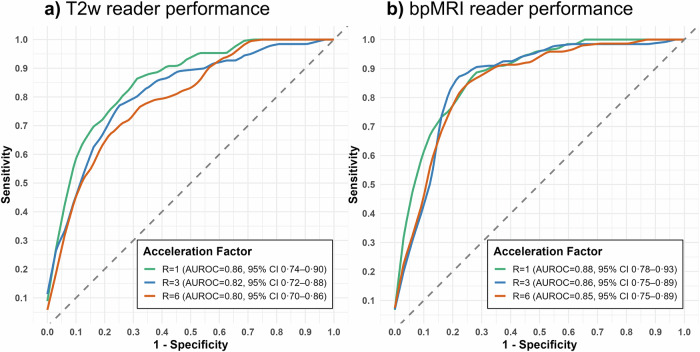
Averaged ROC curves for accelerated and non-accelerated. The mean ROC curves for prostate MRI scans at different acceleration factors (R = 1, R = 3, and R = 6). Each curve reflects the averaged performance across all readers, with the blue, orange, and green lines corresponding to R = 1, R = 3, and R = 6 acceleration factors, respectively: **a** depicts reader performance on T2w, while **b** shows reader performance on complete biparametric MRI (T2w + DWI + ADC). ROC, receiver operating characteristic; T2w, T2-weighted; DWI, diffusion-weighted imaging; ADC, apparent diffusion coefficient; bpMRI, biparametric MRI; AUROC, area under the receiver operating characteristic

Reader diagnostic performance did not differ significantly across acceleration conditions for the T2w protocol (MRMC AUROC comparison across R = 1, R = 3, and R = 6, *p* = 0.08), with AUROCs of 0.86 [95% confidence interval (CI): 0.74–0.90] at R = 1, 0.82 [0.72–0.88] at R = 3, and 0.80 [0.70–0.86] at R = 6 (Fig. 3, Table 3). Pairwise AUROC comparisons revealed a non-significant downward trend with increasing acceleration: Δ = 0.02 [−0.06, 0.10] for R = 1 vs. R = 3 and Δ = 0.04 [−0.05, 0.12] for R = 1 vs. R = 6. For the full biparametric protocol, AUROCs were marginally higher across conditions, 0.88 [0.78–0.93] at R = 1, 0.86 [0.75–0.89] at R = 3, and 0.85 [0.75–0.89] at R = 6, with no significant difference (MRMC AUROC comparison across R = 1, R = 3, and R = 6, *p* = 0.64). Inter-reader agreement, measured via ICCs, declined for T2w imaging at higher acceleration rates (R = 1 vs. R = 6 for T2w imaging in split 1: Δ = −0.07, split 2: Δ = −0.13, and split 3: Δ = −0.39), indicating a potential worse decrease in diagnostic performance on accelerated scans for some readers. Moreover, ICCs remained more stable for bpMRI imaging (R = 1 vs. R = 6 for T2w imaging in split 1: Δ = +0.01, split 2: Δ = +0.05, and split 3: Δ = −0.24), indicating a more variable robustness of reader consensus when combining high accelerations with non-accelerated scans. Complete ICC results are provided in Supplementary Figs. S6, S7, and Table S9.

**Table 3:** Diagnostic and visual metrics of AI reconstructions at R = 3 and R = 6 compared to the non-accelerated R = 1 reference standard

Metric	R = 1	R = 3	R = 6
Diagnostic			
T2w AUROC	0.86 (0.74–0.90)	0.82 (0.72–0.88)	0.80 (0.70–0.86)
bpMRI AUROC	0.88 (0.78–0.93)	0.86 (0.75–0.89)	0.85 (0.75–0.89)
T2w sensitivity	0.93 (0.86–0.98)	0.91 (0.84–0.97)	0.90 (0.82–0.96)
T2w specificity	0.49 (0.43–0.56)	0.51 (0.45–0.58)	0.47 (0.41–0.54)
bpMRI sensitivity	0.97 (0.92–1.00)	0.97 (0.92–1.00)	0.97 (0.92–1.00)
bpMRI specificity	0.45 (0.39–0.51)	0.51 (0.45–0.58)	0.47 (0.41–0.53)
Perceived visual T2w Likert			
Sharpness	2.85 (2.76–2.94)	3.03 (2.94–3.11) (***)	2.94 (2.86–3.02)
Noise	2.78 (2.69–2.88)	2.90 (2.81–2.99) (**)	2.88 (2.80–2.96) (*)
Artifacts	2.97 (2.87–3.07)	3.08 (3.00–3.16)	3.06 (2.97–3.14)
Lesion conspicuity	2.74 (2.63–2.85)	2.84 (2.74–2.94)	2.80 (2.71–2.90)
Overall visual quality	2.74 (2.65–2.83)	2.84 (2.76–2.93)	2.81 (2.73–2.89)
Calculated quality T2w			
SSIM	1.0	0.91 (0.90–0.92)	0.90 (0.89–0.91)
PSNR	Infinite	34.9 (34.2–35.5)	34.2 (33.7–34.8)
RMSE	0.0	19.9 (17.9–21.9)	21.0 (19.1–22.9)
HFEN	0.0	0.27 (0.25–0.28)	0.29 (0.28–0.30)

Metrics are expressed as mean with 95% confidence intervals, where applicable. Perceived visual quality metrics were scored on a 4-point Likert scale (1 = Poor, 4 = Excellent). Higher values of computed metrics, such as structural similarity index and peak signal-to-noise ratio, indicate better structural fidelity and signal clarity, respectively. Lower root mean squared error and high-frequency error norm values represent reduced errors and artifacts. Statistical significance for perceived visual metrics was indicated (* *p* < 0.05, ** *p* < 0.01, *** *p* < 0.001). Data are 95% CIs

*AUROC* area under the receiver operating characteristic, *bpMRI* biparametric magnetic resonance imaging, *SSIM* structural similarity index measure, *PSNR* peak signal-to-noise ratio, *RMSE* root mean squared error, *HFEN* high-frequency error norm, *T2w* T2-weighted

#### PI-RADS assessment

**Fig. 4:**
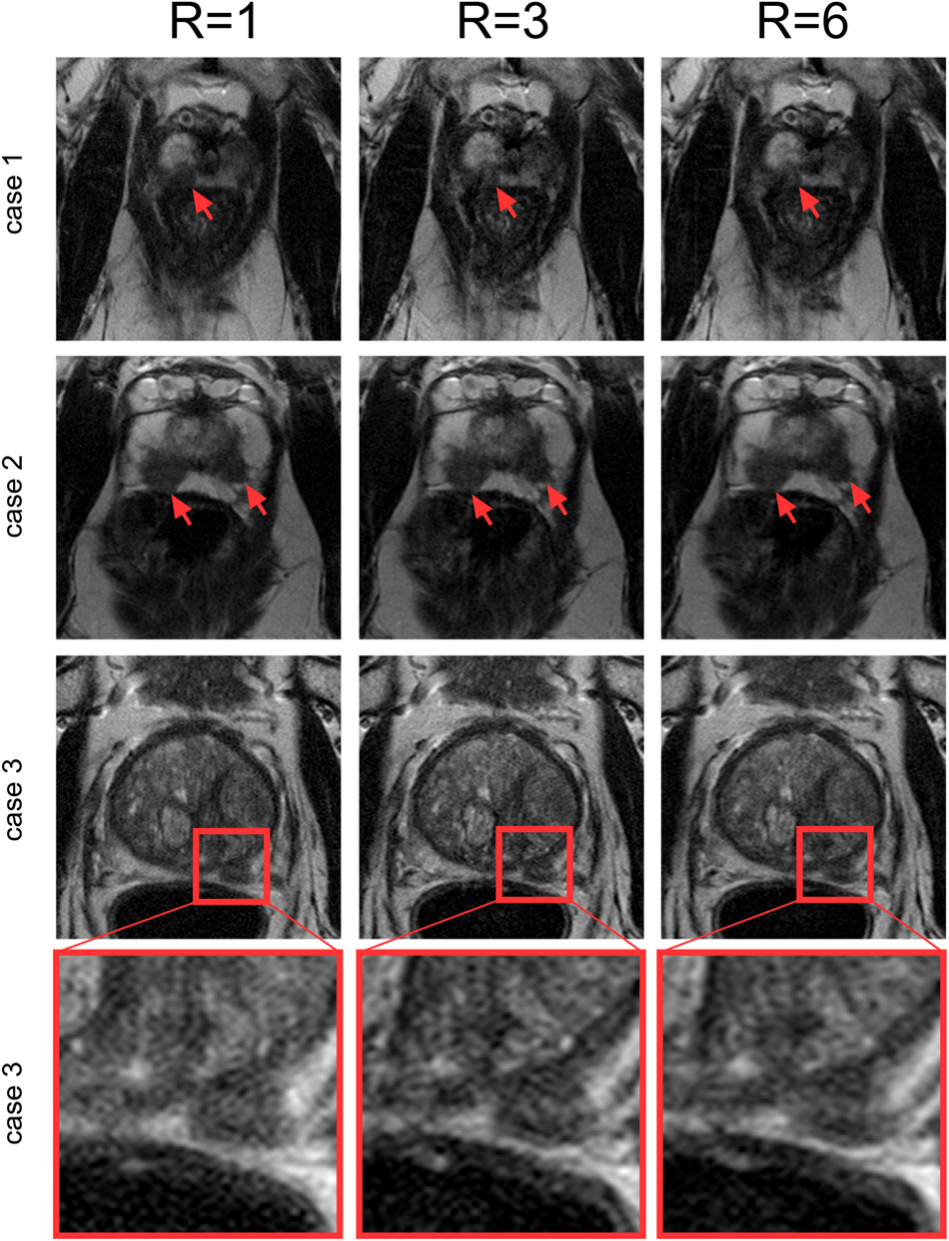
Reconstruction examples. Reconstruction examples at different acceleration factors (R = 1, R = 3, and R = 6). The red arrows indicate a histopathologically proven csPCa lesion. Case 1 shows the reconstructed prostate MRI scan of a 71-year-old patient with a prostate-specific antigen level of 4 ng/mL and a GGG 2 lesion. Case 2 shows the reconstructed prostate MRI scan of a 62-year-old with a prostate-specific antigen level of 14 ng/mL and a left and right GGG 2 lesion. Case 3 shows the reconstructed prostate MRI scan of a 74-year-old with a prostate-specific antigen level of 4 ng/mL and a GGG 4 lesion, zoomed in to demonstrate reconstruction quality. csPCa, clinically significant prostate cancer; GGG, Gleason grade group; MRI, magnetic resonance imaging

Patient-level PI-RADS sensitivity remained stable across all acceleration factors for both T2w and biparametric protocols. For T2w imaging, sensitivity was 0.93 [95% CI: 0.86–0.98] at R = 1, 0.91 [0.84–0.97] at R = 3, and 0.90 [0.82–0.96] at R = 6, while specificity showed minor variation (R = 1: 0.49 [0.43–0.56]; R = 3: 0.51 [0.45–0.58]; R = 6: 0.47 [0.41–0.54]) (Table 3). For the biparametric protocol, sensitivity was uniformly high at 0.97 [0.92–1.00] across all accelerations, with specificities ranging from 0.45 to 0.51. These results indicate preserved diagnostic discrimination despite increased acceleration. Representative reconstructions of three csPCa cases illustrate lesion appearance consistency across acceleration factors (Fig. 4). Reader-specific performance metrics at both patient and lesion levels are detailed in Supplementary Material 2.2.

### Visual performance assessment

#### Reader-perceived image quality

At R = 3, AI-reconstructed images showed improved perceived quality compared to the non-accelerated reference (R = 1). Based on the ordinal mixed-effects model, ratings were significantly higher for sharpness (+0.18 [95% CI: 0.05–0.31], *p* < 0.05) and noise (+0.12 [0.01–0.25], *p* < 0.05), and improved for artifacts (+0.09 [0.01–0.21], *p* = 0.06), lesion conspicuity (+0.10 [0.04–0.25], *p* = 0.05), and overall image quality (+0.10 [0.02–0.22], *p* = 0.06). At R = 6, perceived quality was statistically comparable to R = 1 across all dimensions. These results indicate that reader-perceived image quality is preserved under moderate and high acceleration, with the most pronounced improvements observed at R = 3. Detailed reader-level assessments are presented in Supplementary Material 2.3.

#### Computed image quality

Objective image quality metrics were consistent with reader-perceived assessments, showing minimal degradation at higher acceleration levels (Table 3). At R = 3, the structural similarity index measure was 0.91 [95% CI: 0.90–0.92], and declined slightly to 0.90 [0.89–0.91] at R = 6, indicating strong visual similarity to the non-accelerated reference. Peak signal-to-noise ratio decreased modestly from 34.9 [34.2–35.5] at R = 3 to 34.2 [33.7–34.8] at R = 6. Root mean square error and high-frequency error norm both increased marginally at R = 6, reflecting minor reconstruction discrepancies and subtle degradation of fine detail. These trends are consistent with the reader-assessed improvements in sharpness and noise observed at R = 3. Additional metric distributions are available in Supplementary Material 2.4.

## Discussion

Our findings demonstrated that AI-based reconstruction can substantially accelerate T2w prostate MRI without significantly compromising diagnostic performance. This reduction in scan time translates into shorter examinations, which reduces patient discomfort, lowers the risk of motion artefacts, and enables more efficient use of MRI capacity in healthcare systems facing rising demand. Even at a sixfold acceleration (R = 6; 0:33–1:27 min), we observed no statistically significant decline in csPCa detection compared to the reference standard (R = 1; 3:18–8:40 min), although a downward trend in performance was noted (*p* = 0.08). This result suggests that higher acceleration levels may approach a threshold where diagnostic accuracy begins to degrade. At R = 3, perceived image quality surpassed that of the fully sampled standard, consistent with known denoising effects associated with AI-based reconstruction [23].

This study builds upon important prior work that demonstrates the feasibility of AI-driven acceleration [24]. A systematic review by Reinhardt et al focused on studies looking at AI-accelerated prostate MRI. Their review showed gains in image quality and feasibility in smaller or simulated cohorts, but performance variances underscored the need for larger, more robust studies [24]. Compared with the studies in the systematic review, our results are based on more expert readers (i.e., 8 vs. max 4 readers [3]), more data (i.e., 120 vs. max 109 cases [25]), and we performed our study on multi-coil k-space data. Moreover, Gassenmaier et al applied deep learning acceleration to T2w prostate MRI in 30 patients, reporting improved image quality and preserved PI-RADS classifications, though without biopsy-confirmed accuracy or multi-reader assessment [26]. Similarly, Rastogi et al evaluated a denoising algorithm on 45 retrospective T2w scans (~ R = 3 protocol) with nine readers with 4 ± 6.5 years experience (vs. 12.1 ± 4.3 years in our study), showing trends toward preserved diagnostic utility but without histopathological validation [5]. Building on this foundation, our study provides large-scale, multi-reader, biopsy-validated evidence that diagnostic accuracy is preserved even with substantial acceleration. Importantly, we assessed performance within a clinically representative biparametric protocol, where combining accelerated T2w with non-accelerated DWI proved robust in retaining diagnostic value.

Although previous studies have shown that AI-based models can generate high-quality MRI reconstructions [3, 4, 24–27], many of these efforts relied on computed similarity metrics, which do not always correlate with diagnostic performance [6]. We therefore report similarity metrics alongside reader assessments as complementary endpoints. A key concern is the potential for hallucination AI-generated features that resemble plausible anatomy but do not reflect actual tissue, thereby altering lesion appearance and increasing the risk of both false positives and false negatives [4, 6]. To mitigate this risk, we employed a physics-informed, model-based reconstruction framework designed to constrain output to data-consistent anatomical structures [10, 28, 29]. Our training and implementation code is publicly available to support transparency and further validation (https://github.com/NKI-AI/direct [30] and https://github.com/Quintin1995/direct-with-averages).

This study has limitations. First, generalizability may be restricted because all data were acquired on a single vendor platform and acceleration was simulated retrospectively. Although undersampling was retrospective, the patterns used are easily applicable on modern scanners, supporting clinical feasibility. Second, workflow conditions differed from clinical routine: radiologists used an online reading platform, and the reference standard reconstruction omitted vendor-specific preprocessing to ensure uniformity across acceleration factors, which may reduce comparability with hospital PACS and clinical-grade images. Third, the definition of negative cases relied on PI-RADS ≤ 2 and biopsy without long-term follow-up, and image quality assessment was limited to T2w sequences with PI-QUALv2–derived criteria. Nonetheless, the high negative predictive value of PI-RADS for csPCa (90–97%) [31] supports the validity of the approach. Fourth, our consecutive clinical cohort contained relatively few transition-zone lesions (Supplementary Table S3), which limited zone-specific comparisons of diagnostic performance between peripheral- and transition-zone disease, where T2-weighted imaging plays a key role in PI-RADS assessment. Lastly, we observed substantial reader-to-reader variability for PI-RADS threshold-based sensitivity and specificity, particularly for specificity. This variability is potentially related to the number of point annotations made by the readers (Supplementary Table S8). Such variability must be considered when interpreting the generalizability of performance metrics derived from MRMC studies.

AI-driven reconstruction enabled a sixfold acceleration of T2w prostate MRI (0:33–1:27 min) without a statistically significant reduction in PCa detection, while preserving perceived image quality. However, a downward trend suggests diagnostic performance may degrade at higher accelerations than six and warrants further prospective evaluation.

## Supplementary information

ELECTRONIC SUPPLEMENTARY MATERIAL

## Abbreviations

ADC: Apparent diffusion coefficient map
AI: Artificial intelligence
AUROC: Area under the receiver operating characteristic
csPCa: Clinically significant prostate cancer
DWI: Diffusion-weighted imaging
GGG: Gleason grade group
MRMC: Multi-reader multi-case
NYU: New York University
PI-RADS: Prostate imaging-reporting and data systems
T2w: T2-weighted
