# Supplementary material for: Diagnostic assessment of artificial intelligence reconstruction on accelerated prostate MRI: a retrospective, paired, multi-reader multi-case study
Source: Eur Radiol. 2026 Apr 2;36(8):6440–9. doi: 10.1007/s00330-026-12479-7 (PMC13341839; doi:10.1007/s00330-026-12479-7)

Diagnostic assessment of artificial intelligence reconstruction on accelerated prostate MRI: a retrospective, paired, multi-reader multi-case study

ELECTRONIC SUPPLEMENTARY MATERIAL

<u>Table of Contents</u>

1. Supplementary methods 1

1.1. Study design 1

1.1.1. Power analysis 1

1.1.2. Experimental analysis 2

1.2. AI model specifications 2

1.2.1. Training data 3

1.2.2. K-space undersampling 3

1.2.3. Data augmentations 3

1.2.4. Model architecture 3

1.2.5. Parameter optimization 4

1.2.6. Loss function 4

1.2.7. Reconstruction post-processing 4

1.3. Reader study design 5

1.3.1. Study cohort and split-plot design 5

1.3.2. Reading schedule and blinding protocol 5

1.3.3. Implementation and workflow optimization 6

1.4. Reader study evaluation 6

1.4.1. Diagnostic reference standard 6

1.4.2. Visual reference standard 6

1.4.3. Diagnostic quality assessment 7

1.4.4. Visual quality assessment 9

2. Supplementary results 11

2.1. Participating readers 11

2.2. Reader-specific diagnostic performance 11

2.3. Reader-specific perceived visual quality assessment 12

2.4. Computed image quality 12

2.5. Reader agreement analysis 12

Supplementary references 14

Supplementary tables 16

Supplementary figures 25

1. Supplementary methods

The methodology applied in the main manuscript is explained in more detail in the following sections. The order of the sections corresponds to the order in which they were used during the study. Subsection ***1.1*** describes the study design. Subsection ***1.2*** provides the AI model specifications and training methodology. Subsection ***1.3*** describes the reader study in more detail. Subsection ***1.4*** describes the reader study evaluation.

1.1. Study design

To assess reader and case variations in csPCa suspicion scores over the acceleration factors (R=1, 3, 6), a partially paired multi-reader multi-case (MRMC) factorial study design was constructed. Prior to the analysis, a power analysis estimated the number of readers and cases required for enough statistical power.

1.1.1. Power analysis

The iMRMC (available at: [https://github.com/DIDSR/iMRMC]) sizing module was employed for statistical power calculation. The resulting number of cases and readers were validated in Nancy Obuchowski's power table for MRMC studies.[1] The MRMC model calculates statistical power based on pre-specified variance components, including the number of readers, case distribution, level of statistical significance (alpha), and effect size. Unlike Obuchowski's method, the iMRMC model supports split-plot design analysis, allowing for more complex experimental setups.

We compared the baseline bpMRI readings with the accelerated bpMRI readings to determine the effect size. The baseline bpMRI performance, representing real variance between readers, was derived from 20 radiologists with an average AUC of 0.85. For the accelerated bpMRI readings, we simulated the performance of 20 radiologists, assuming a difference in AUC of 0.05 to reflect performance variations between conventional and accelerated conditions.

In line with existing literature, the significance level was set at 0.05, with a ratio of 70:30 for negative to positive cases.[2] We tested the impact of split design on the radiologists' workload using the iMRMC tool's paired-reading paired-cases setting.

A power analysis was performed using the iMRMC tool to assess the ability to detect differences in diagnostic performance variations between baseline and accelerated readings. This analysis accounted for configurations of readers, cases, and split groups, simulating performance based on a negative-to-positive case ratio of 3:1. ***Table S1*** provides an overview of the tested configurations and corresponding power estimates, demonstrating that the study achieved sufficient statistical power (≥0.80) across scenarios with three or more readers and 120 cases.

1.1.2. Experimental analysis

Our study compared the diagnostic accuracy of AI-based reconstructions against conventional, non-accelerated scans. Our analysis assessed whether the performance of non-accelerated MRI was superior to that of AI-based reconstructed scans.

With the MRMC analysis, we performed a superiority test. The average reader performance measured as the area under the receiver operating characteristic (AUROC) was computed using the diagonal average method, which preserves the area under the curve.[3] Superiority is concluded if the AUROC difference between the two sets of readings is greater than 0.

The iMRMC sizing module uses the F-test to calculate statistical power based on MRMC components of variance for the given number of cases, their class distribution, the number of readers, and the effect size. In this study, the computation flow was in accordance with the workflow of Gallas et al.[4] The test was performed with the paired-reading paired-cases setting, simulating different reader scenarios to ensure robust power estimates.

1.2. AI model specifications

For this study, an existing state-of-the-art AI reconstruction model was trained on the publicly available NYU prostate k-space dataset.

1.2.1. Training data

The training data comprises the publicly available prostate k-space dataset from NYU (fastMRI).[5] It included 312 patients referred for clinical biparametric prostate MRI on 3T systems. Raw data comprised multi-coil acquisitions and were scanned with three averages: the first and third averages sampling the odd lines, and the second samples even lines. The reference standards were constructed by combining all three averages in the k-space domain.

1.2.2. K-space undersampling

During the training phase, fully sampled MRI data was subjected to retrospective undersampling at various acceleration factors: 2, 4, 6, and 8. A specific fraction of the fully-sampled central region was retained for each acceleration factor 16%, 8%, 5.33%, and 4%, respectively. These retained data were essential for estimating and predicting sensitivity maps.

The experiments employed three predefined retrospective undersampling schemes, as suggested in the referenced study.[6] These schemes included Equispaced, Random (Uniform), and Gaussian Cartesian trajectories, which were retrospectively applied to generate and impose undersampling patterns on the training k-space data to achieve the desired acceleration. ***Figure S1*** depicts an example of the resulting undersampling patterns.

1.2.3. Data augmentations

To improve the model's generalization capabilities during training, we applied random data augmentations on the fully sampled measurements, including cropping, flipping, and rotation. Cropping augmentations were performed in the image domain (before projecting back to the k-space domain), whilst flipping and rotation augmentations were applied in the k-space domain.

1.2.4. Model architecture

The model architecture incorporated a state-of-the-art deep learning-based MRI reconstruction algorithm, the variable Splitting Half-quadratic ADMM algorithm for Reconstruction of Inverse Problems (vSHARP).[7] This architecture featured T=14 ADMM

optimization steps and utilized two-dimensional U-Nets with four scales and 32 filters at the initial scale for the ADMM denoising step. The data consistency step of ADMM was set to 10 gradient descents. Additionally, a 2D U-Net with four scales and 32 filters at the first scale was employed for the sensitivity module of vSHARP, with all other hyperparameters following the guidelines of the original publication.

1.2.5. Parameter optimization

Model parameters were optimized using the Adam optimizer, with ϵ = 10^−8 and (β1, β2) = (0.99, 0.999). The learning rate was initially scaled linearly from 0.00104 to 0.003 over the first 1000 iterations. Subsequently, a learning rate scheduler was implemented, reducing the learning rate by a factor of 0.9 every 40,000 training iterations. The experiments ran for approximately 200,000 iterations on two A6000 RTX GPUs, with each GPU handling a batch size of two slices of multi-coil k-space data. The best model weights for checkpointing were selected based on performance evaluations using validation data from the NYU prostate data.

1.2.6. Loss function

We employed a dual-domain loss calculation to train the model, analyzing both the image and frequency domains. Specifically, this approach integrated a combination of mean absolute error (MAE) and structural similarity index measure (SSIM) loss in the image domain, complemented by a normalized MAE loss in the frequency domain. Each component of the loss function was equally weighted.

1.2.7. Reconstruction post-processing

To ensure the reconstructions meet clinical MRI standards, they underwent post-processing to align the lesion locations with those of the original DICOM and match the physical dimensions and field of view of the T2w image with DWI and ADC. Firstly, the images' x, y, and z dimensions were flipped to match clinical orientation. Secondly, k-space zero-filling was applied, resulting in image space interpolation that doubled the image space resolution, aligning the reconstructions with standard imaging protocols. At last, the images were center-

cropped to maintain a consistent field of view with the vendor-processed images. These adjustments ensured that lesions initially segmented on clinical MRI—validated through a biopsy for csPCa—aligned correctly with the AI-based reconstructions.

1.3. Reader study design

1.3.1. Study cohort and split-plot design

The reader study was structured using a three-session, three-part stratified split-plot design to evaluate the diagnostic accuracy of T2-weighted (T2w) AI reconstructions. The study cohort consisted of 120 unique prostate MRI patients collected from the BLINDED CENTER (BLINDED CENTER) between May 10, 2022, and January 28, 2024. Each case was represented under three acceleration conditions: R=1 (non-accelerated), R=3 (moderately accelerated), and R=6 (highly accelerated), resulting in a total of 360 cases. These cases were divided into three splits containing 40 unique patients to ensure balanced and independent diagnostic evaluations. The stratification ensured an equitable distribution of csPCa and non-csPCa cases across splits, providing a representative diagnostic challenge.

1.3.2. Reading schedule and blinding protocol

Each radiologist was assigned to only one split, ensuring no overlap or duplication. Within their assigned split, radiologists reviewed all 40 patients under the three acceleration conditions (R=1, R=3, and R=6), evaluating a total of 120 cases. The split structure was divided into three sessions to ensure robust diagnostic evaluations and minimize potential recall bias. The same 40 patients were presented during each session but with a different stratified mixture of acceleration conditions. The sequence of cases and acceleration levels was randomized within each session, and a four-week washout period was enforced between sessions to minimize memory effects and maintain independent case assessments. This stratified and randomized session structure ensured comprehensive exposure to each acceleration condition while reducing bias.

1.3.3. Implementation and workflow optimization

Before starting the reading phase, each radiologist received a guide detailing the annotation workflow hosted on grandchallenge.org. This guide included instructions on using various tools within the platform, such as navigation, zooming, and windowing features. Additionally, to ensure familiarity with the system and consistency in interpretation, radiologists practiced on cases not part of the primary study cohort. This preparatory phase was crucial for standardizing the evaluation process across all participants, ensuring that each radiologist approached the study with a uniform understanding of the assessment criteria and platform functionality. ***Figure S2*** shows the reading interface and the two-stage annotation workflow.

1.4. Reader study evaluation

1.4.1. Diagnostic reference standard

The diagnostic reference standard in this study defined csPCa as pathology confirmation with Gleason Grade Group (GGG) ≥ 2. The included patients underwent targeted biopsy after suspicion of csPCa at MRI (PI-RADSv2 ≥ 3)[8]; see the STARD diagram in ***Supplementary material 5.1***. In addition to targeted biopsy, a portion of patients received systematic biopsy. The reference standard was based on all available biopsy information (targeted and systematic). The lesion-level reference standard was based on the clinical csPCa segmentation for targeted biopsy with required changes based on the biopsy findings (i.e., a new segmentation was made by an expert radiologist based on the systematic biopsy csPCa findings).

1.4.2. Visual reference standard

The image-space reference standard was generated using the conventional root sum of squares (RSS) method, which was applied to the prostate k-space data. This process accounted for multiple averages, slices, coils, and encoding dimensions, including frequency and phase encoding. The BLINDED CENTER testing data was processed in the following steps:

1. **Combining averages:** Each average covers half of k-space, even or odd lines. To integrate the data from multiple averages, we combined them as follows: the first and third averages were summed and then averaged. This result was then combined with the second average. This method ensures that the final image includes all acquired k-space lines.
2. **Inverse Fourier transform:** An image from the acquired k-space in the frequency domain was obtained using an inverse Fourier transform.
3. **RSS calculation**: To generate the reference standard image, the combined k-space data from all coils was first transformed into image space using an inverse Fourier transform (IFT), performed for each slice individually. The root sum of squares (RSS) method was then applied, combining the coil images into a single composite image by calculating the square root of the sum of the squared magnitudes of each coil's image:

$$RSS = \sqrt{\Sigma_i |X_i|^2}$$

   Here X_i represents the image from the i-th coil. This method integrates data from all coils into a single high-SNR image by leveraging their combined signal strength while effectively reducing noise.

The RSS approach ensured that the resulting reference standard image aligned with clinical imaging standards. Combining coil data across slices and averages produces a high-quality image reconstruction (R=1), the reference standard for evaluating accelerated AI reconstructions.

1.4.3. Diagnostic quality assessment

The primary outcome was a diagnostic performance comparison between T2w AI reconstructions and non-accelerated MRI. The diagnostic assessment is performed using an MRMC analysis and performance metrics on PI-RADS diagnosis. The MRMC analysis involved comparing multiple readers evaluating multiple cases.[9-11] This analysis makes a trade-off between the number of readers, the workload per reader, and the available splits, [12] and the proportion of diseased:non-diseased. These variables, in combination with the inter-

and intra-reader variability from the literature, provide a power estimation, see ***Supplementary material 1.1***.

The MRMC analysis extends the conventional ROC comparison at the patient level. In our analysis, we will calculate the AUROC for each radiologist for the standard and AI-based reconstructed imaging protocols using the assigned csPCa suspicion scores (ranging from 0 for no csPCa suspicion to 100 for certain csPCa presence). This approach allows us to assess the individual diagnostic accuracy of each radiologist for each protocol and aggregate these AUROCs to evaluate the overall performance and reliability of the standard versus AI-accelerated protocols for all readers. By analyzing these AUROCs collectively, we can draw conclusions about the relative effectiveness of each imaging method in detecting csPCa, considering variations in individual radiologist performance. This analysis aimed to assess the superiority of non-accelerated MRI (R=1) over accelerated MRI (R=3 and R=6) regarding diagnostic performance.

Two statistical tests were conducted: one comparing the AUROCs of non-accelerated (R=1) versus AI reconstructed (R=3) MRI and another comparing R=1 versus (R=6. The objective was to ascertain whether significant differences exist, confirming or refuting the efficacy of AI-based acceleration in clinical diagnostics. For the comparison, we use the public FDA-approved software iMRMC v4.0.3 software (Division of Imaging, Diagnostics, and Software Reliability, FDA/CDRH/OSEL)[4] and the MRMCaov R studio package[13]. The diagonal average for average ROC analysis, which is area-preserving, was used to average the individual ROC values together.[3]

A patient-level PI-RADS sensitivity and specificity analysis complemented the diagnostic quality assessment. The PI-RADS analysis was based on radiologists' point marked and lesion-level PI-RADS scoring during reading. On a patient level, the highest PI-RADS score was considered and compared with the histopathology reference standard to calculate the patient-level sensitivity and specificity (see **Tables S4 and S6**). On the lesion level, the lesion marker point set by the reader was compared with the histopathological segmentation. The histopathological segmentation was used for targeted biopsy and updated with systematic

histopathological findings. In one case, the segmentation could not be updated because no lesion could be identified, although there was a positive systematic biopsy finding. This case was excluded from the lesion analysis. In the analysis, the number of true positives, false positives, true negatives, and false negatives were counted and used to calculate the lesion-level sensitivity and specificity (see **Tables S5 and S7**).[2] A lesion marker point was considered a true positive (TP) if it was located within 5 mm of the histopathological segmentation, a threshold chosen to account for radiologists' typical margin of error and variability in histopathological segmentation due to tissue deformation.[2] To prevent inflation of TP counts, only one was recorded if multiple markers were placed within the same lesion. Conversely, a false positive (FP) was defined as a lesion marker point outside the 5 mm boundary of any histopathological segmentation. In cases with no histopathological segmentation, the absence of any marker points was classified as a true negative (TN). Conversely, if no marker points were placed in the presence of a histopathological segmentation indicating a lesion, this was recorded as a false negative (FN).

1.4.4. Visual quality assessment

Radiologists evaluated the visual quality of MRI images on artifacts, sharpness, noise, lesion conspicuity, and overall visual quality. In line with two previous visual comparison studies, a four-point Likert scale was used in which a score of 0 indicated no diagnostic assessment possible due to hampered readability, and a score of 4 indicated perfect diagnostic quality.[14,15] The perceived visual quality scores of R=1 and accelerated (R=3, R=6) images were compared with an ordinal mixed-effect model. This approach accounts for the hierarchical structure of our data (radiologists and cases). Unlike more straightforward non-parametric tests, the mixed-effects model incorporates random effects, effectively providing more accurate estimates and handling variability among radiologists and cases. This visual assessment was designed to detect significant differences in visual quality between non-accelerated and AI-accelerated (R=3 or R=6) cases, producing ten p-values: five comparing visual metrics R=1 versus R=3 and another five for R=1 versus R=6. The objective was to

assess whether significant differences exist, confirming or refuting the efficacy of AI reconstruction acceleration in terms of visual quality.

In addition, to evaluate the visual quality of the AI reconstructions, we utilized several established image quality metrics: the SSIM, Peak Signal-to-Noise Ratio (PSNR), Root Mean Squared Error (RMSE), and High-Frequency Error Norm (HFEN). These metrics were chosen for their relevance in image reconstruction and ability to capture different aspects of image quality. SSIM is widely used in reconstruction model training and evaluation due to its ability to assess visual similarity, making it a crucial metric for comparing the fidelity of accelerated images to their non-accelerated counterparts. PSNR is another commonly used metric measuring signal-to-noise ratio, providing insight into the overall image quality and noise levels. RMSE, one of the more traditional metrics, quantifies the average error magnitude between the images, offering a straightforward assessment of reconstruction accuracy. HFEN, focusing on high-frequency components, is particularly valuable for prostate MRI as it emphasizes preserving fine details. These metrics were compared between R=1 and accelerated (R=3, R=6) images, and the mean values were reported with 95% confidence intervals.

2. Supplementary results

2.1. Participating readers

Eight readers participated in the study, completing the study between May 2024 and October 2024. All readers had extensive experience with prostate MRI reading, ranging from 7 to 20 years. Specifically, readers had the following years of experience: Reader 1: 8 years, Reader 2: 10 years, Reader 3: 13 years, Reader 4: 11 years, Reader 5: 18 years, Reader 6: 20 years, Reader 7: 10 years, and Reader 8: 7 years. Five readers were based in the Netherlands, two in the United States, and one in the United Kingdom.

2.2. Reader-specific diagnostic performance

Figure S3 shows the diagnostic performance per reader over each acceleration condition. In addition, ***Tables S4-S7*** provide the sensitivity and specificity for patient-level PI-RADS assessments across readers and acceleration factors for T2w-only protocols. Sensitivity remained consistently high across all readers and accelerations, with an overall slight decrease from R=1 (0.93 sensitivity) to R=3 (0.91 sensitivity) and R=6 (0.90 sensitivity). Notably, readers 1, 4, 6, and 7 maintained perfect sensitivity (1.00 sensitivity) across all accelerations, while reader 8 exhibited a noticeable decline at R=6 (0.62 sensitivity). Specificity showed more variability, ranging from 0.10 to 0.75 across readers. At R=3, patient-level specificity improved slightly (0.49 vs 0.51 specificity), with reader 8 achieving the highest specificity (0.88 specificity). However, patient-level specificity declined slightly at R=6 (0.49 vs 0.48 specificity), with some readers (e.g., readers 1, 6, and 7) showing values below 0.40 specificity. The variation in specificity was related to the number of point annotations made by the readers, shown in **Table S8**. These results emphasize robust diagnostic sensitivity across acceleration factors while highlighting variability in reader-specific specificity.

2.3. Reader-specific perceived visual quality assessment

A reader-specific perceived visual quality performance can be viewed in ***Figure S4***. While the difference in performance per acceleration is more challenging to observe in the average scores, this is clearer in the reader-specific results.

2.4. Computed image quality

The computed image quality metrics for T2w AI reconstructions across acceleration factors R=3 and R=6 are shown in ***Figure S5.*** Metrics such as SSIM and PSNR demonstrated consistent values across the test dataset, with SSIM scores of 0.91 (95% CI: 0.90–0.92) and 0.90 (95% CI: 0.89–0.91) and PSNR scores of 34.9 (95% CI: 34.2–35.5) and 34.2 (95% CI: 33.7–34.8) for R=3 and R=6, respectively. RMSE and HFEN, which measure reconstruction error and high-frequency detail retention, also showed stable distributions, with RMSE increasing slightly from 19.9 (95% CI: 17.9–21.9) at R=3 to 21.0 (95% CI: 19.1–22.9) at R=6, and HFEN values of 0.27 (95% CI: 0.25–0.28) and 0.29 (95% CI: 0.28–0.30). Notably, the SSIM results obtained in this study closely align with the training phase results despite being evaluated on a completely independent dataset from a different institute. While data properties between the training and test datasets share similarities, these findings highlight the robustness of the reconstruction model across diverse institutional datasets.

2.5. Reader agreement analysis

To quantify reader consistency across acceleration conditions, we computed the intraclass correlation coefficient (ICC) for suspicion scores within each reader split and acceleration factor. ICC values were derived using a one-way random-effects model for average measures. ***Supplementary Figures S6*** (T2-only) and ***S7*** (bpMRI) show that agreement was consistently high in splits 2 and 3, while split 1 exhibited a progressive decline in agreement with increasing acceleration. ***Supplementary Table S9*** provides the full ICC results, including 95% confidence intervals, the number of cases, and the number of readers per configuration.

Although AI-based acceleration preserved average diagnostic performance, reader agreement varied substantially across reader groups and acceleration levels, potentially related to the number of point annotations made by the readers, shown in **Table S8**. The observed decline in inter-reader ICC, particularly in split 1 at R=6, indicates reduced reproducibility under high acceleration. This suggests that even if AUROC remains stable on average, the diagnostic reliability across observers may be compromised. Such variability must be considered when interpreting the generalizability of performance metrics derived from MRMC studies.

Supplementary references

[1] Obuchowski NA. Sample size tables for receiver operating characteristic studies. AJR Am J Roentgenol. 2000 Sep;175(3):603-8. doi: 10.2214/ajr.175.3.1750603. PMID: 10954438.

[2] Saha A, Bosma JS, Twilt JJ, et al. Artificial intelligence and radiologists in prostate cancer detection on MRI (PI-CAI): an international, paired, non-inferiority, confirmatory study. Lancet Oncol. 2024 Jul;25(7):879-887. doi: 10.1016/S1470-2045(24)00220-1. Epub 2024 Jun 11. PMID: 38876123.

[3] Chen W, Samuelson FW. The average receiver operating characteristic curve in multireader multicase imaging studies. Br J Radiol. 2014 Aug;87(1040):20140016. doi: 10.1259/bjr.20140016. Epub 2014 Jun 2. PMID: 24884728; PMCID: PMC4112395.

[4] Gallas BD, Bandos A, Samuelson FW, Wagner RF. A framework for random-effects ROC analysis: biases with the bootstrap and other variance estimators. Commun Stat Theory Methods. 2009;38(15):2586-2603. doi:10.1080/03610920802610084.

[5] Tibrewala R, Dutt T, Tong A, et al. FastMRI Prostate: A public, biparametric MRI dataset to advance machine learning for prostate cancer imaging. Sci Data. 2024;11:404. doi:10.1038/s41597-024-03252-w.

[6] Yiasemis G, Sánchez CI, Sonke JJ, Teuwen J. On retrospective k-space subsampling schemes for deep MRI reconstruction. Magn Reson Imaging. 2024 Apr;107:33-46. doi: 10.1016/j.mri.2023.12.012.

[7] Yiasemis G, Moriakov N, Sonke JJ, Teuwen J. vSHARP: Variable Splitting Half-quadratic ADMM algorithm for reconstruction of inverse-problems. Magn Reson Imaging. 2024 Oct 24:110266. doi: 10.1016/j.mri.2024.110266. Epub ahead of print. PMID: 39461485.

[8] Turkbey B, Rosenkrantz AB, Haider MA, et al. Prostate Imaging Reporting and Data System Version 2.1: 2019 Update of Prostate Imaging Reporting and Data System Version 2. Eur Urol. 2019 Sep;76(3):340-351. doi: 10.1016/j.eururo.2019.02.033. Epub 2019 Mar 18. PMID: 30898406.

[9] McKinney SM, Sieniek M, Godbole V, et al. International evaluation of an AI system for breast cancer screening. Nature. 2020 Jan;577(7788):89-94. doi: 10.1038/s41586-019-1799-

6. Epub 2020 Jan 1. Erratum in: Nature. 2020 Oct;586(7829):E19. doi: 10.1038/s41586-020-2679-9. PMID: 31894144.

[10] Rodriguez-Ruiz A, Lång K, Gubern-Merida A, et al. Stand-Alone Artificial Intelligence for Breast Cancer Detection in Mammography: Comparison With 101 Radiologists. J Natl Cancer Inst. 2019 Sep 1;111(9):916-922. doi: 10.1093/jnci/djy222. PMID: 30834436; PMCID: PMC6748773.

[11] Ehteshami Bejnordi B, Veta M, Johannes van Diest P, et al. Diagnostic Assessment of Deep Learning Algorithms for Detection of Lymph Node Metastases in Women With Breast Cancer. JAMA. 2017 Dec 12;318(22):2199-2210. doi: 10.1001/jama.2017.14585. PMID: 29234806; PMCID: PMC5820737.

[12] Chen W, Gong Q, Gallas BD. Paired split-plot designs of multireader multicase studies. J Med Imaging (Bellingham). 2018 Jul;5(3):031410. doi: 10.1117/1.JMI.5.3.031410. Epub 2018 May 17. PMID: 29795776; PMCID: PMC5956142.

[13] Smith BJ, Hillis SL. Multi-reader multi-case analysis of variance software for diagnostic performance comparison of imaging modalities. Proc SPIE Int Soc Opt Eng. 2020 Feb;11316:113160K. doi: 10.1117/12.2549075. Epub 2020 Mar 16. PMID: 32351258; PMCID: PMC7190386.

[14] Johnson PM, Tong A, Donthireddy A, et al. Deep Learning Reconstruction Enables Highly Accelerated Biparametric MR Imaging of the Prostate. J Magn Reson Imaging. 2022 Jul;56(1):184-195. doi: 10.1002/jmri.28024. Epub 2021 Dec 7. PMID: 34877735; PMCID: PMC9170839.

[15] Gassenmaier S, Afat S, Nickel D, Mostapha M, Herrmann J, Othman AE. Deep learning-accelerated T2-weighted imaging of the prostate: Reduction of acquisition time and improvement of image quality. Eur J Radiol. 2021 Apr;137:109600. doi: 10.1016/j.ejrad.2021.109600. Epub 2021 Feb 15. PMID: 33610853.

Supplementary tables

Table S1: The iMRMC power analysis tool was used to perform the power analysis for various study configurations, including split groups, readers, and cases per reader. Negative-to-positive case ratios were fixed at 3:1, and power was calculated to evaluate the adequacy of study designs, targeting ≥ 0.80.

Cases, n	neg:pos	Split groups	readers, n	Cases per reader (Total)	Readers per split group	Power
120	3:1	2	4	60 (180)	2	0.95
120	3:1	2	6	60 (180)	3	0.98
120	3:1	3	3	40 (120)	2	0.80
120	3:1	3	6	40 (120)	2	0.94
124	3:1	4	4	30 (90)	1	0.80
124	3:1	4	8	30 (90)	2	0.94

Table S2: DWI acquisition parameters of the high b-value DWI and ADC within the BLINDED CENTER testing cohort.

Protocol	DWI	DWI
View	Axial	Axial
Siemens MR type	Skyra (64%)	Prisma (36%)
Voxel size (mm x mm)	3.51x3.51	1.75x1.75
Field of view (mm x mm)	200x200	200x200
Slice thickness (mm)	3	3
Matrix size	114x114	114x114
Coils, n	10-30*	10-30*
slices, n	26-38*	26-38*
b-values	50, 500, 1000 & calc1400	50, 500, 1000 & calc1400
Averages, n	1, 3, 6 & 6	1, 3, 5 & 5
Phase encoding steps, n	99	114
Scan time (min:sec) [IQR]	3:37 [3:21-3:42]	2:54 [2:33-2:58]

* Indicates the data range.

Table S3: This table provides an overview of the number of PZ and TZ lesions per split and the median lesion size for each. Five patients had two lesions. In one PZ case of split 2, the segmentation could not be updated because no lesion could be identified, although a positive systematic biopsy finding was found. This case was excluded from the lesion analysis and the volume calculation. One outlier in PZ (16.94 cc) and TZ (10.25 cc) lesions in Split 1 substantially increased the standard deviation. For this reason, lesion volumes are also summarized using the median and IQR.

Split	PZ (cc median ± IQR)	TZ (cc median ± IQR)
1	7 (0.65 ± 1.93 cc)	3 (0.71 ± 9.75 cc)
2	9 (0.29 ± 0.33 cc)	0
3	9 (0.51 ± 0.63 cc)	1 (0.95 cc)

Table S4: The patient-level PI-RADS sensitivity and specificity performance per reader and acceleration condition on T2-only protocols. Sensitivity remained high across all readers and acceleration factors, with an overall slight decrease from R=1 (0.93 sensitivity) to R=3 (0.91 sensitivity) and R=6 (0.90 sensitivity). Specificity showed more variability, with values ranging from 0.10 to 0.75 across readers. Notably, readers 1, 4, 6, and 7 maintained perfect sensitivity (1.00) across all accelerations, whereas reader 8 showed a noticeable decline in sensitivity at R=6 (0.62 sensitivity). The mean sensitivity and specificity with 95% CI were obtained from 10000 bootstrap resamples of patients with replacement while keeping the participating readers fixed were 0.93 [0.86–0.98] and 0.50 [0.43–0.56], 0.91 [0.84–0.97] and 0.51 [0.45–0.58], and 0.90 [0.82–0.96] and 0.48 [0.41–0.54] at respectively R=1, R=3, and R=6.

Split	Reader	Baseline (R=1)		R=3		R=6	
		Sensitivity	Specificity	Sensitivity	Specificity	Sensitivity	Specificity
1	1	1.00	0.13	1.00	0.10	1.00	0.10
1	2	1.00	0.55	0.89	0.45	0.89	0.52
1	3	0.89	0.71	0.89	0.74	0.78	0.68
2	4	0.88	0.38	1.00	0.56	1.00	0.47
2	5	0.75	0.50	0.75	0.62	0.88	0.50
3	6	1.00	0.53	0.88	0.38	1.00	0.44
3	7	1.00	0.41	1.00	0.38	1.00	0.34
3	8	0.88	0.75	0.88	0.88	0.62	0.75
All		0.93	0.49	0.91	0.51	0.90	0.47

Table S5: The lesion-level PI-RADS sensitivity and specificity performance per reader and acceleration condition on T2w-only protocols. Sensitivity remained relatively stable across acceleration factors, with values of 0.67, 0.60, and 0.60 for R=1, R=3, and R=6, respectively. Specificity showed no meaningful differences, with values of 0.36, 0.35, and 0.33 across the same conditions. Reader-specific variability was more apparent than differences between acceleration factors, with some readers (e.g., reader 7) achieving higher specificity values (up to 0.80 specificity at R=3). In contrast, others (e.g., reader 1) had lower specificity values (as low as 0.04 across all conditions). The mean sensitivity and specificity with 95% CI were obtained from 10000 bootstrap resamples of patients with replacement while keeping the participating readers fixed were 0.67 [0.56–0.77] and 0.36 [0.31–0.41], 0.60 [0.49–0.71] and 0.35 [0.30–0.40], and 0.60 [0.49–0.71] and 0.33 [0.28–0.37] at respectively R=1, R=3, and R=6.

**Split**	**Reader**	**Baseline (R=1)**		**R=3**		**R=6**	
		**Sensitivity**	**Specificity**	**Sensitivity**	**Specificity**	**Sensitivity**	**Specificity**
1	1	0.70	0.06	0.60	0.04	0.60	0.04
1	2	0.70	0.41	0.60	0.35	0.70	0.41
1	3	0.70	0.58	0.70	0.64	0.60	0.58
2	4	0.67	0.24	0.56	0.38	0.67	0.29
2	5	0.56	0.42	0.56	0.51	0.67	0.43
3	6	0.70	0.45	0.50	0.29	0.50	0.35
3	7	0.60	0.32	0.70	0.24	0.60	0.22
3	8	0.70	0.67	0.60	0.80	0.50	0.65
All		0.67	0.36	0.60	0.35	0.60	0.33

Table S6: The patient-level PI-RADS sensitivity and specificity performance per reader and acceleration condition on bpMRI protocols. Sensitivity remained consistently high across all acceleration factors (R=1, R=3, and R=6), with an overall value of 0.97 for R=1 and R=3 and 0.97 at R=6. Specificity showed minor variations across acceleration conditions, increasing slightly from 0.45 at R=1 to 0.51 at R=3, before decreasing to 0.47 at R=6. Reader-specific variability was more pronounced, with some readers (e.g., reader 1) demonstrating low specificity (0.03–0.06) across all acceleration conditions, while others (e.g., reader 8) achieved higher specificity values (up to 0.84 at R=3). The mean sensitivity and specificity with 95% CI were obtained from 10000 bootstrap resamples of patients with replacement while keeping the participating readers fixed were 0.97 [0.92–1.0] and 0.45 [0.39–0.51], 0.97 [0.92–1.0] and 0.51 [0.45–0.58], and 0.97 [0.92–1.0] and 0.47 [0.41–0.53] at respectively R=1, R=3, and R=6.

Split	Reader	Baseline (R=1)		R=3		R=6	
		Sensitivity	Specificity	Sensitivity	Specificity	Sensitivity	Specificity
1	1	1.00	0.03	1.00	0.03	1.00	0.06
1	2	1.00	0.68	1.00	0.65	0.89	0.61
1	3	1.00	0.65	1.00	0.58	1.00	0.77
2	4	0.88	0.41	1.00	0.59	1.00	0.53
2	5	0.88	0.44	0.75	0.62	0.88	0.44
3	6	1.00	0.31	1.00	0.34	1.00	0.31
3	7	1.00	0.41	1.00	0.44	1.00	0.28
3	8	1.00	0.69	1.00	0.84	1.00	0.75
All		0.97	0.45	0.97	0.51	0.97	0.47

Table S7: The lesion-level PI-RADS sensitivity and specificity performance per reader and acceleration condition on bpMRI protocols. Sensitivity remained stable across acceleration factors, with values of 0.76, 0.74, and 0.77 for R=1, R=3, and R=6, respectively. Specificity was low overall and showed minimal variation across acceleration factors, with values of 0.32, 0.36, and 0.32 for R=1, R=3, and R=6, respectively. The mean sensitivity and specificity with 95% CI were obtained from 10000 bootstrap resamples of patients with replacement while keeping the participating readers fixed were 0.76 [0.66–0.85] and 0.32 [0.27–0.37], 0.74 [0.64–0.84] and 0.36 [0.31–0.41], and 0.77 [0.67–0.86] and 0.32 [0.27–0.37] at respectively R=1, R=3, and R=6.

Split	Reader	Baseline (R=1)		R=3		R=6	
		**Sensitivity**	**Specificity**	**Sensitivity**	**Specificity**	**Sensitivity**	**Specificity**
1	1	0.80	0.01	0.80	0.01	0.70	0.02
1	2	0.80	0.55	0.70	0.57	0.80	0.53
1	3	0.70	0.53	0.90	0.56	0.80	0.67
2	4	0.78	0.29	0.78	0.45	0.89	0.41
2	5	0.56	0.34	0.44	0.49	0.67	0.35
3	6	0.80	0.26	0.70	0.27	0.80	0.25
3	7	0.80	0.30	0.80	0.33	0.70	0.20
3	8	0.80	0.63	0.80	0.77	0.80	0.67
All		0.76	0.32	0.74	0.36	0.77	0.32

Table S8: The number of point annotations made per reader for T2W and bpMRI questions and the number of correct and false point annotations. The total number of point annotations by reader 1 is n=527, double the second highest amount of reader 7 with n=246. The majority of point annotations made during reading were false positives.

		Point-annotations		
**Reader**	**Split**	**Total**	**Correct**	**False**
**T2w questions**				
Reader 1	1	244	20	224
Reader 2	1	94	21	73
Reader 3	1	64	20	44
Reader 4	2	123	17	106
Reader 5	2	85	16	69
Reader 6	3	94	17	77
Reader 7	3	123	19	104
Reader 8	3	50	18	32
**bpMRI questions**				
Reader 1	1	283	25	258
Reader 2	1	73	24	49
Reader 3	1	68	24	44
Reader 4	2	107	22	85
Reader 5	2	95	15	80
Reader 6	3	111	23	88
Reader 7	3	123	26	97
Reader 8	3	57	24	33

Table S9: Intraclass correlation coefficient (ICC) estimates for continuous suspicion scores, stratified by reader split, acceleration factor (R), and imaging protocol (T2-only and biparametric MRI [bpMRI]). ICC values were computed using a one-way random-effects model for average measures to quantify inter-reader reliability. Each entry includes the 95% confidence interval in brackets.

Split	R	T2 ICC [95% CI]	bpMRI ICC [95% CI]	N Cases	N readers
3	1	0.81 [0.68-0.89]	0.80 [0.67-0.89]	40	3
3	3	0.78 [0.63-0.88]	0.71 [0.51-0.84]	40	3
3	6	0.74 [0.56-0.85]	0.81 [0.68-0.89]	40	3
2	1	0.82 [0.67-0.91]	0.85 [0.72-0.92]	40	2
2	3	0.80 [0.62-0.89]	0.84 [0.69-0.91]	40	2
2	6	0.69 [0.42-0.84]	0.90 [0.82-0.95]	40	2
1	1	0.61 [0.35-0.78]	0.56 [0.26-0.75]	40	3
1	3	0.46 [0.10-0.70]	0.45 [0.08-0.69]	40	3
1	6	0.22 [-0.32-0.56]	0.32 [-0.14-0.62]	40	3

Supplementary figures

Figure S1: Three examples of retrospective undersampling patterns for a k-space slice were used during the reconstruction model's training. These patterns undersample at a factor of R=4 and represent three random possibilities of undersampling. Besides these patterns of R=4 undersampling, other patterns with R=2, R=6, and R=8 were also employed to make the model robust to different undersampling factors. During training, the clinical utility of the patterns was not considered; the employed undersampling pattern in the testing dataset did consider the clinical utility.

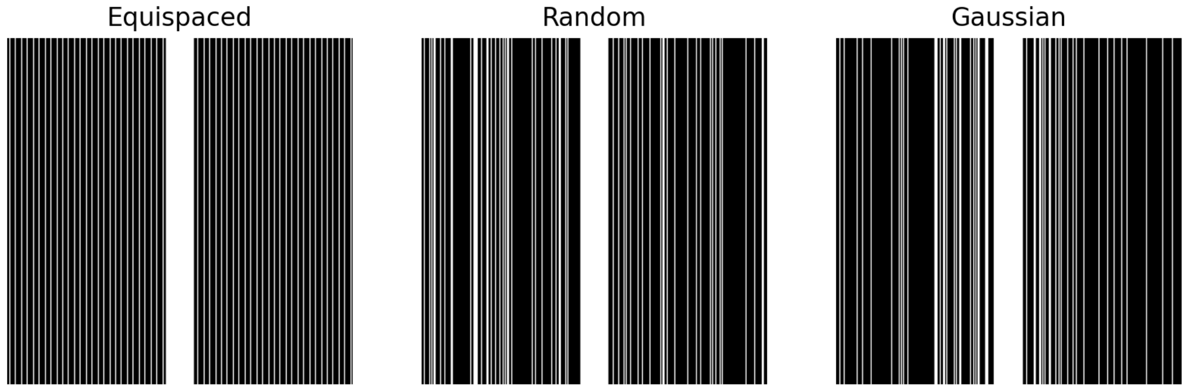

Figure S2: The reader study was conducted on the online reader platform GrandChallenge. The reading interface is depicted, and the two-stage annotation workflow is in which the T2w questions (visual and diagnostic) were first answered, followed by the bpMRI questions (diagnostic). The answers were saved between the stages, and no information on non-accelerated DWI and ADC could be used to answer the T2w questions.

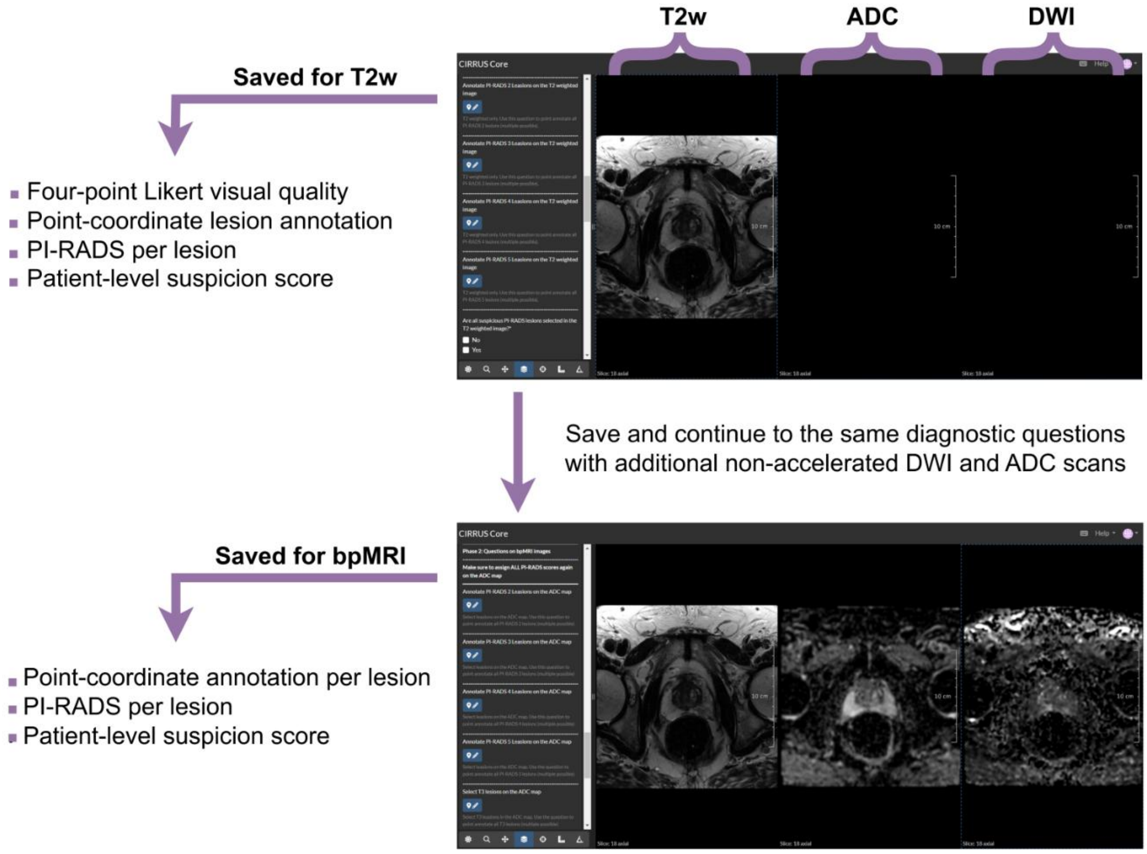

Figure S3: A depiction of the AUROC area under the receiver operating characteristic (AUROC) per reader over each acceleration condition on T2w scans. The average T2w AUROC with 95% confidence was 0.86 [0.74-0.90], 0.82 [0.72-0.88], and 0.80 [0.70-0.86] for R=1, R=3, and R=6, respectively.

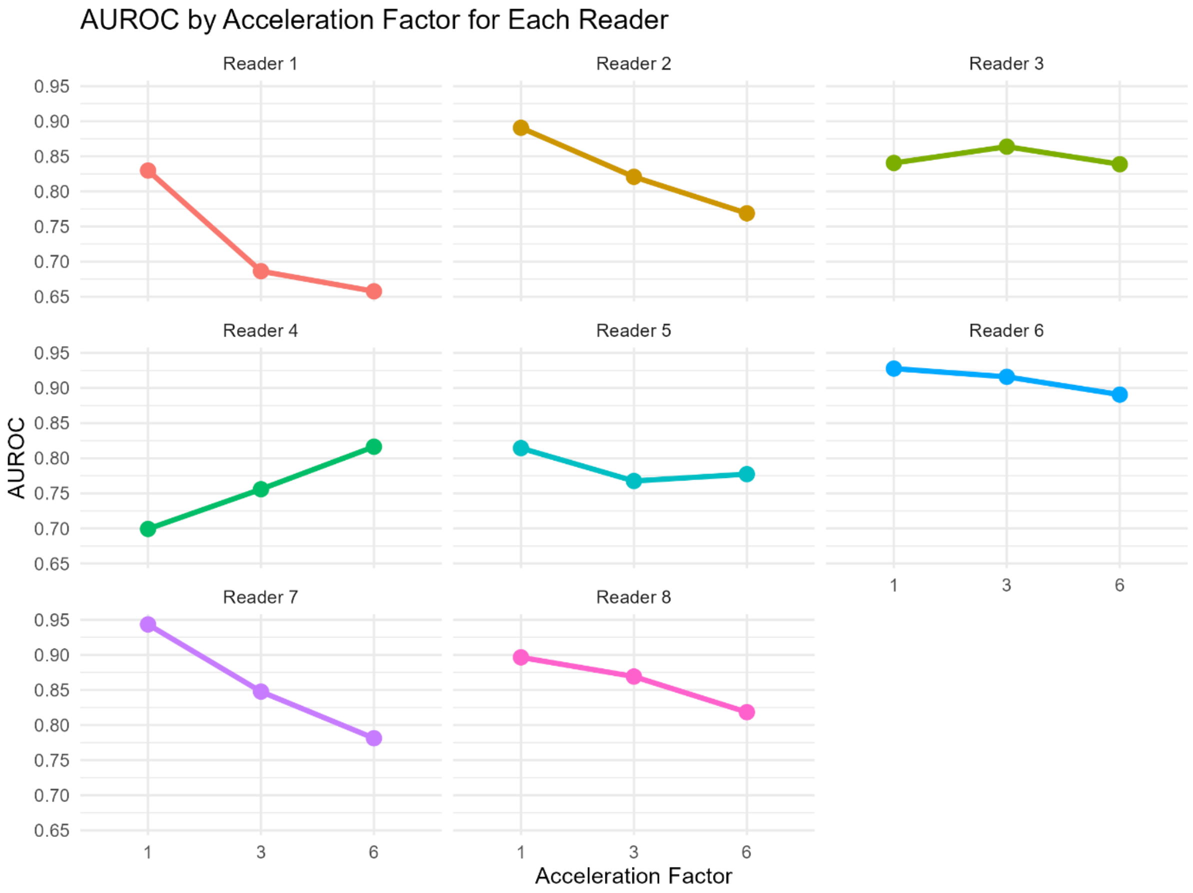

Figure S4: Histograms showing the distribution of perceived visual quality scores for overall T2-image quality, categorized by reader. The x-axis represents the quality scores, which range from poor to excellent, while the y-axis indicates the frequency of these scores as assigned by the readers. Notably, the non-accelerated data R=1 (in red) generally receives lower scores compared to the R=3 data (in green), with this trend being particularly evident for reader 2. The T2w perceived visual quality scores were 2.85 [95% CI: 2.76–2.94], 3.03 [95% CI: 2.94–3.11], and 2.94 [95% CI: 2.86–3.02] for sharpness, 2.78 [95% CI: 2.69–2.88], 2.90 [95% CI: 2.81–2.99], and 2.88 [95% CI: 2.80–2.96] for noise, 2.97 [95% CI: 2.87–3.07], 3.08 [95% CI: 3.00–3.16], and 3.06 [95% CI: 2.97–3.14] for artifacts, 2.74 [95% CI: 2.63–2.85], 2.84 [95% CI: 2.74–2.94], and 2.80 [95% CI: 2.71–2.90] for lesion conspicuity, and 2.74 [95% CI: 2.65–2.83], 2.84 [95% CI: 2.76–2.93], and 2.81 [95% CI: 2.73–2.89] for overall visual quality for respectively R=1, R=3, and R=6. The 95% CIs were calculated using 10.000 bootstraps with case and reader resamples.

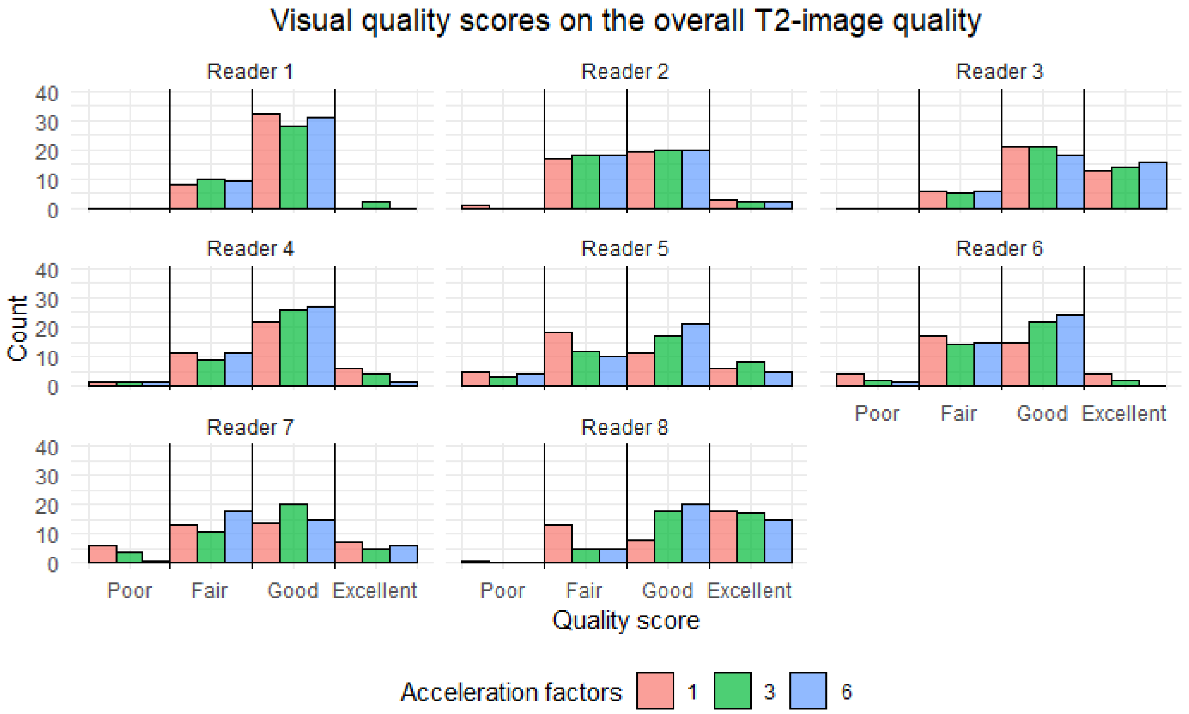

Figure S5: Visual quality metrics distribution of AI-based reconstructions and R=3 and R=6. For SSIM and PSNR, a higher value represents better image similarity and signal-to-noise ratios. In comparison, RMSE and HFEN have a lower value, representing better overall image similarity and better retention of high-frequency details. The SSIM scores were 0.91 (0.90–0.92) and 0.90 (0.89–0.91), the PSNR scores were 34.9 (34.2–35.5) and 34.2 (33.7–34.8), the RMSE scores were 19.9 (17.9–21.9) and 21.0 (19.1–22.9), and the HFEN scores were 0.27 (0.25–0.28) and 0.29 (0.28–0.30) for respectively R=3 and R=6.

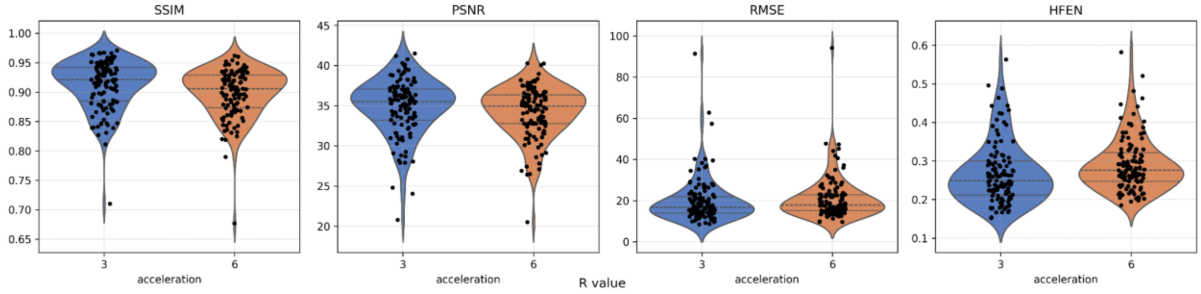

Figure S6: Intraclass correlation coefficient (ICC) estimates for radiologist-assigned suspicion scores on AI-reconstructed T2-weighted images only, stratified by reader split (1–3) and acceleration factor (R=1, R=3, R=6). ICC values were computed using a one-way random-effects model for average measures, reflecting inter-reader agreement within each split. Vertical lines denote 95% confidence intervals.

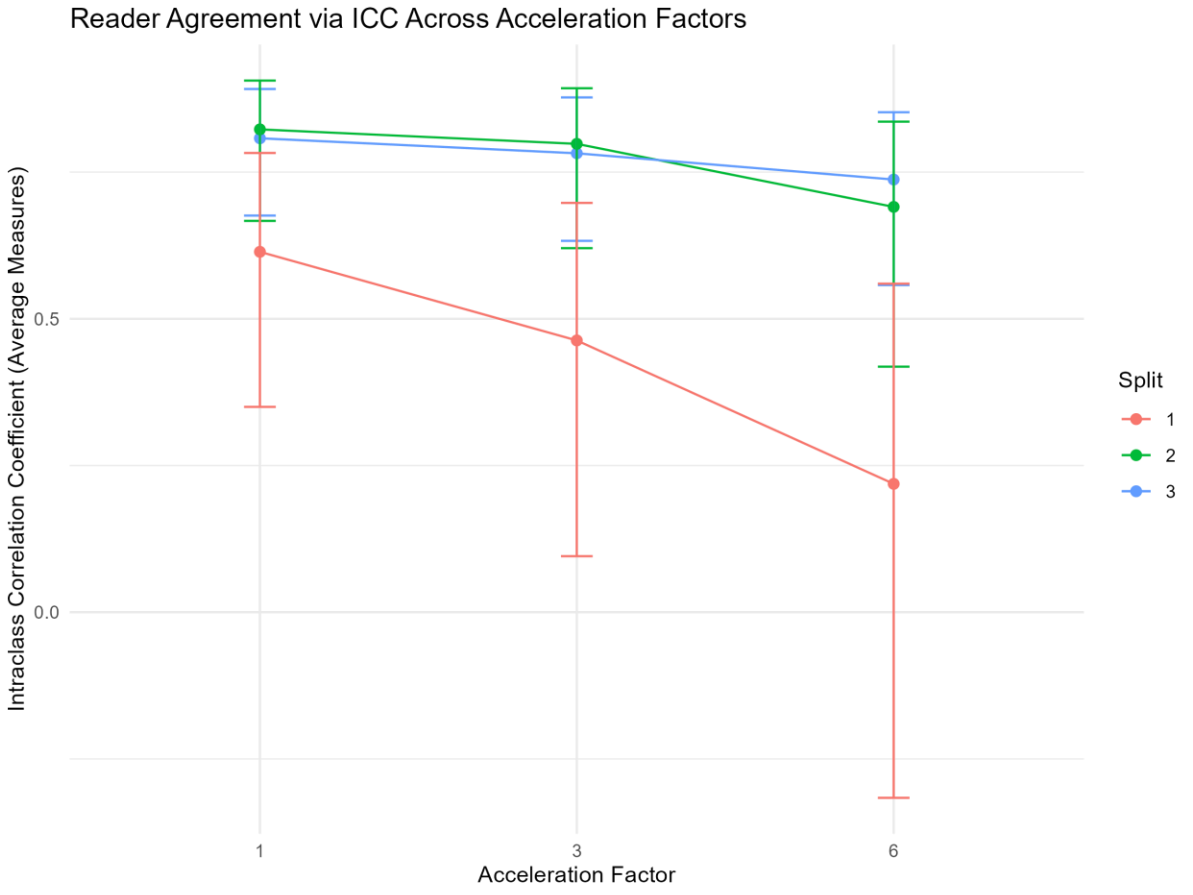

Figure S7: Intraclass correlation coefficient (ICC) estimates for radiologist-assigned suspicion scores on biparametric MRI (T2 + DWI) images, stratified by reader split (1-3) and acceleration factor (R=1, R=3, R=6). ICC was calculated using a one-way random-effects model for average measures. Confidence intervals (95%) are shown as vertical error bars.

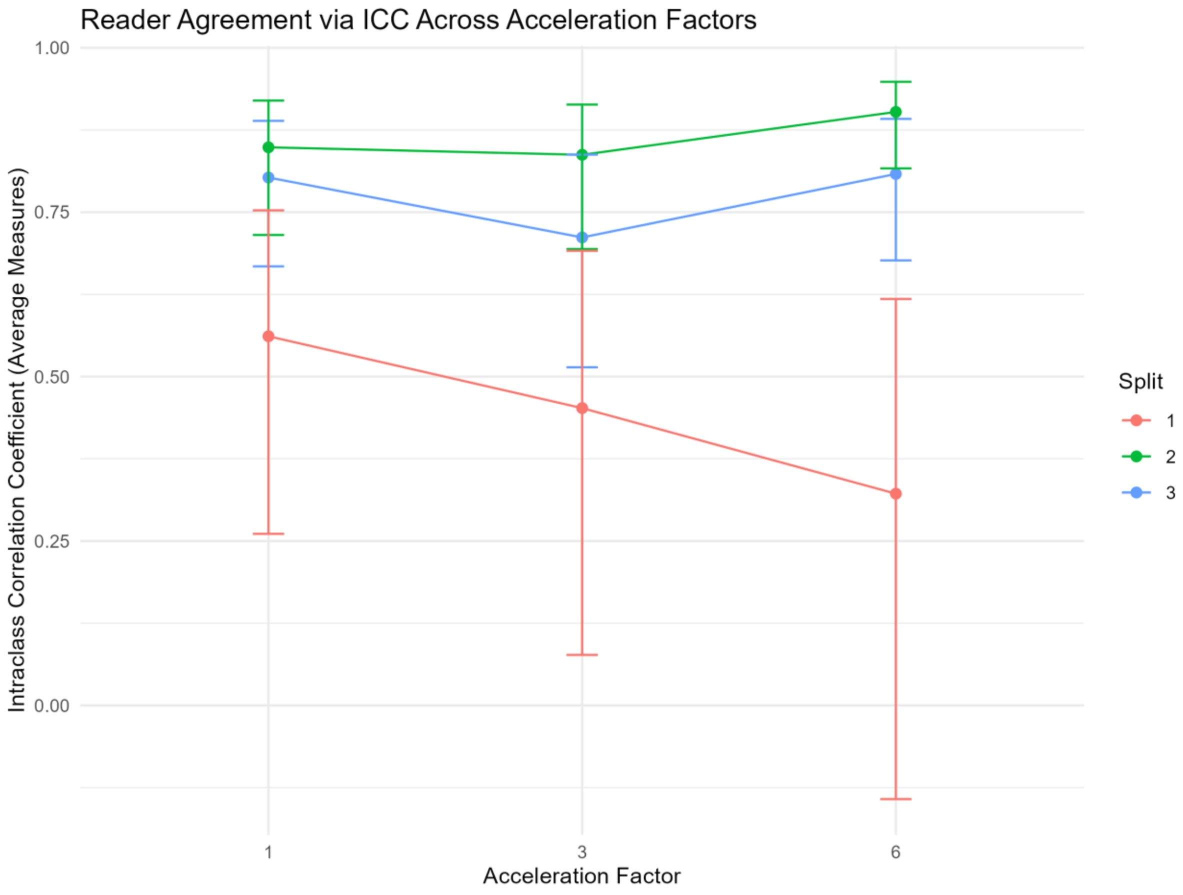

Supplement: Supplementary file 1 — ELECTRONIC SUPPLEMENTARY MATERIAL [file 330_2026_12479_MOESM1_ESM.pdf]
